# Significant Improvement of Puncture Accuracy and Fluoroscopy Reduction in Percutaneous Transforaminal Endoscopic Discectomy With Novel Lumbar Location System: Preliminary Report of Prospective Hello Study: Erratum

**DOI:** 10.1097/01.md.0000494757.87873.e2

**Published:** 2016-09-09

In the article “Significant Improvement of Puncture Accuracy and Fluoroscopy Reduction in Percutaneous Transforaminal Endoscopic Discectomy With Novel Lumbar Location System: Preliminary Report of Prospective Hello Study”,^1^ which appeared in Volume 94, Issue 49 of *Medicine*, several typos appeared in the original article. The article has since been corrected online.
